# Polarized Regulation of Glycogen Synthase Kinase-3β Is Important for Glioma Cell Invasion

**DOI:** 10.1371/journal.pone.0081814

**Published:** 2013-12-03

**Authors:** Qifei Zou, Ying Hou, Feng Shen, Yizheng Wang

**Affiliations:** 1 Eastern Hepatobiliary Surgery Hospital, Second Military Medical University, Shanghai, China; 2 Laboratory of Neural Signal Transduction, Institute of Neuroscience, Chinese Academy of Sciences, Shanghai, China; The Ohio State University, United States of America

## Abstract

Glioma malignancy greatly depends on its aggressive invasion. The establishment of cell polarity is an important initial step for cell migration, which is essential for cell-directional translocation. However, our understanding of the molecular mechanisms underlying cell polarity formation in glioma cell invasion remains limited. Glycogen synthase kinase-3 (GSK-3) has a critical role in the formation of cell polarity. We therefore investigated whether localized GSK-3β, a subtype of GSK-3, is important for glioma cell invasion. We reported here that the localized phosphorylation of GSK-3β at the Ser9 (pSer9-GSK-3β) was critical for glioma cell invasion. Scratching glioma cell monolayer up-regulated pSer9-GSK-3β specifically at the wound edge. Inhibition of GSK-3 impaired the cell polarity and reduced the directional persistence of cell migration. Consistently, down-regulation of GSK-3α and 3β by specific small interfering RNAs inhibited glioma cell invasion. Over-expressing wild-type or constitutively active forms of GSK-3β also inhibited the cell invasion. These results indicated the polarized localization of GSK-3 regulation in cell migration might be also important for glioma cell migration. Further, EGF regulated both GSK-3α and 3β, but only pSer9-GSK-3β was enriched at the leading edge of scratched glioma cells. Up- or down-regulation of GSK-3β inhibited EGF-stimulated cell invasion. Moreover, EGF specifically regulated GSK-3β, but not GSK-3α, through atypical PKC pathways. Our results indicated that GSK-3 was important for glioma cell invasion and localized inhibition of GSK-3β was critical for cell polarity formation.

## Introduction

Glioblastoma multiform is the most common and lethal brain tumor, which results largely from its highly invasive property [1]. Although considerable progress has been made in surgical and radiation treatment for glioma patients in the past decades, the clinical outcome has been disappointing with median survival time not exceeding 15 months [2]. This is partially due to our poor understanding of the molecular mechanisms underlying the aggressive invasion of glioma cells.

When cells migrate, distinctive steps of cell locomotion are sequentially carried out, including morphological polarization, membrane extension, formation of cell-substratum attachment and contractile force, cell body traction, and finally release of attachment [3]. Among these steps, the establishment of cell polarity is an important initial step, since such spatial asymmetry of cytoskeleton and cellular organelle is essential for generation of intracellular force providing power for cell-directional translocation [4]. Cell polarity is generally defined as a status that the cytoskeleton and cellular organelles are spatially arranged in an asymmetric way [5-7]. Among multiple forms of cell polarity, the lost of the planar cell polarity (PCP) was associated with tumor progression [6]. Tumor cells invade into surrounding tissues in a directional way rather than a random way, suggesting an underlying cell polarity formation and maintenance [8-10]. However, the mechanism for the establishment of cell polarity in migrating tumor cells is still elusive. The GSK-3β, an important regulator for various biological processes [11,12], has been shown to be essential for the cell polarity formation in astrocytes and neurons [13,14]. In astrocytes, localized inhibition of GSK-3β was critical for the orientation of microtubule-organizing center (MTOC) of cells at the wound edge in scratched astrocyte monolayers, suggesting that GSK-3β is possibly involved in astrocyte migration. We thus asked whether the GSK-3β-dependent cell polarity was important for glioma cell invasion. 

In this report, we provided evidences that GSK-3 was important for serum- or EGF-stimulated glioma cell invasion. When glioma cells stimulated with serum or EGF, GSK-3β was regulated through its localized inhibition, characterized by the increased phosphorylation at the Ser9 of GSK-3β (pSer9-GSK-3β) at the leading edge of migrating glioma cells. Furthermore, the localized inhibition of GSK-3β was important for cell polarity formation and cell invasion. Although down-regulation of GSK-3α also suppressed cell invasion, the phosphorylation at the Ser21 of GSK-3α (pSer21-GSK-3α) was not regulated in an asymmetric way and likely had different upstream signals as GSK-3β. Collectively, our results supported that GSK-3 was important for glioma cell invasion and that localized regulation of GSK-3β was critical.

## Results

### Polarized GSK-3β inhibition was necessary for the formation of glioma cell polarity

**Figure 1:**
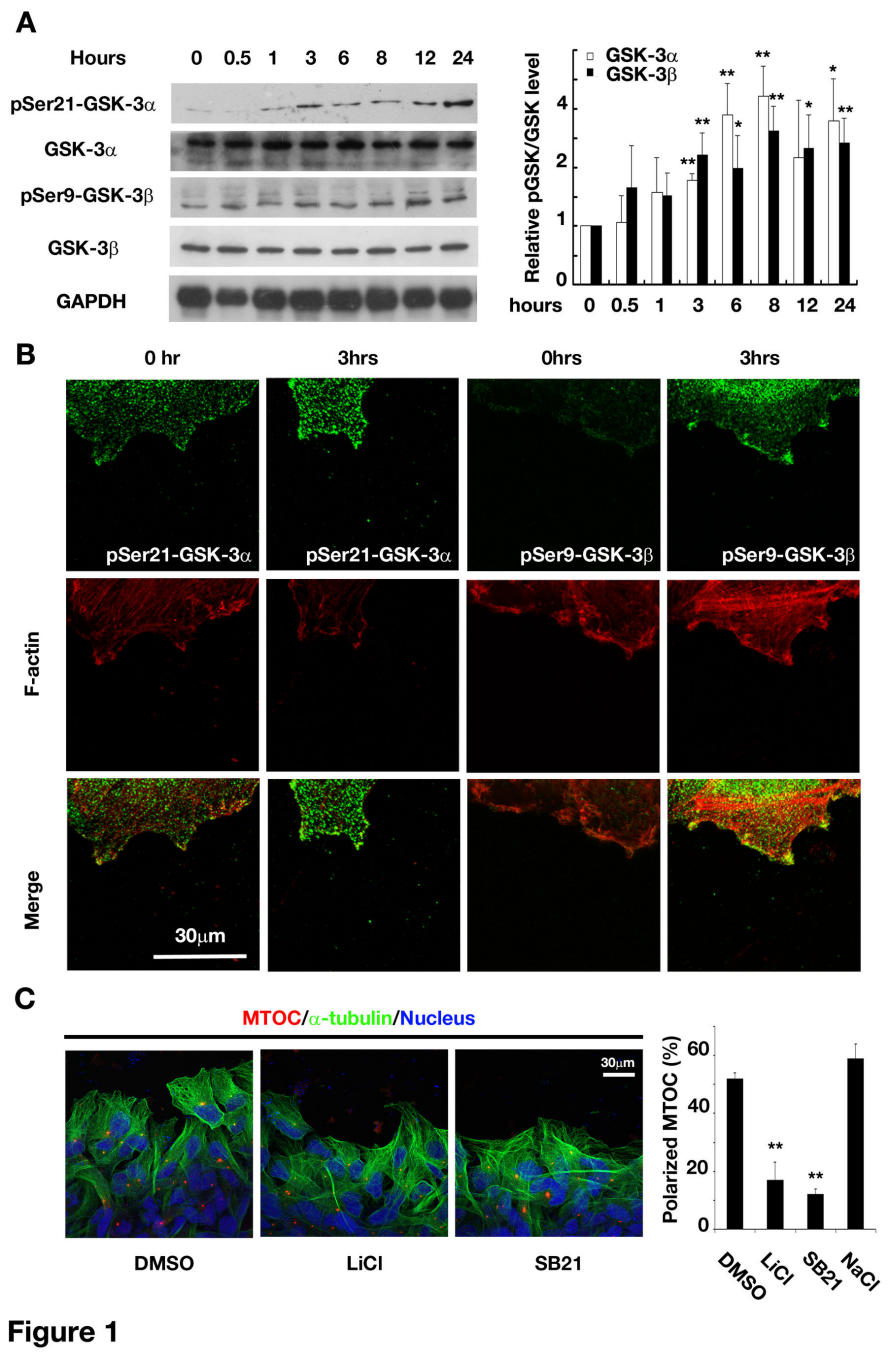
Localized inactivation of GSK-3β was associated with the formation of cell polarity. (A) Up-regulation of pSer-GSK-3 during the wound recovery of U251 cells. *Left*: representative western blot results of U251 cell lysates collected at indicated time points after scratching. *Right*: statistics of p-Ser-GSK-3 levels normalized to GSK-3 levels and compared with the value at 0hr. *P<0.05, ** *P*<0.01 versus the value of pGSK-3/GSK-3 at 0hr. (B) Location of increased pGSK-3 staining in the wound recovery of U251 cells at 0 hr or 3 hrs after scratching. Green: anti-pSer21-GSK-3α or anti-pSer9-GSK-3β; Red: rhodamine phalloidin. *Original magnification, 400×*.(C) MTOC mis-orientation in U251 caused by GSK-3 inhibitors. *Left*: representative images of MTOC in different treatments. Green: anti-α-tubulin; Red: anti-pericentrin; Blue: Hoechst 33258. *Original magnification, 400×*. *Right*: quantification of centrosome polarization as described in *Materials and Methods* in U251 with or without GSK-3β inhibitors. ** *P*<0.01 versus DMSO. LiCl 20mM, SB216763, 20μM, NaCl, 20 mM. Data were mean ± SD of three independent experiments.

To study whether GSK-3 was involved in glioma cell migration, we first examined the stepwise change in the levels of pSer21-GSK-3α and pSer9-GSK-3β, phosphorylation sites important for their inactivation [11], in glioma cell monolayers in response to a scratching wound stimulus. We found that both phosphorylated GSK-3α and 3β levels were greatly increased, whereas the total level of GSK-3α and 3β was not changed, suggesting a decrease in their kinase activities (Figure 1A). Immuno-staining of phosphorylated GSK-3α and GSK-3β showed that pSer9-GSK-3β mainly was at the leading edge of the cells located at the wound margin, whereas pSer21-GSK-3α inhibition evenly distributed (Figure 1B). We did not find asymmetric localization of total GSK-3 by staining GSK-3α and 3β (data not shown). Therefore, inhibition of GSK-3βwas found only at the scratching side, towards which the cells would migrate. We then assayed the MTOC, a structure indicating the direction of microtubule rearrangement and cell movement. Normally, the microtubule organizing center (MTOC) will be re-oriented to a position between the leading edge and the nucleus during directional cell migration. The MTOC orientation renders cell polarity formation contributing to polarized delivery of membrane precursors and actin regulatory factors toward the leading edge. Cells in the first row showing the centrosome located in front of the nucleus and in the 120° sector facing the wound were defined as properly oriented. The MTOC was present in the quadrant that was in front of the nucleus towards the wound in about 50% of control cells 9hrs after scratching (Figure 1C). However, application of LiCl and SB216763, specific GSK-3 inhibitors, caused MTOC randomly distributed around the nucleus (Figure 1C). These results are consistent with an explanation that inhibition of GSK-3β disrupted the cell polarity. Therefore, our results suggested that polarized inactivation of GSK-3β was necessary for the establishment of cell polarity.

### Inhibiting GSK-3 reduced the directional persistence and locomotion speed of glioma cell migration

**Figure 2:**
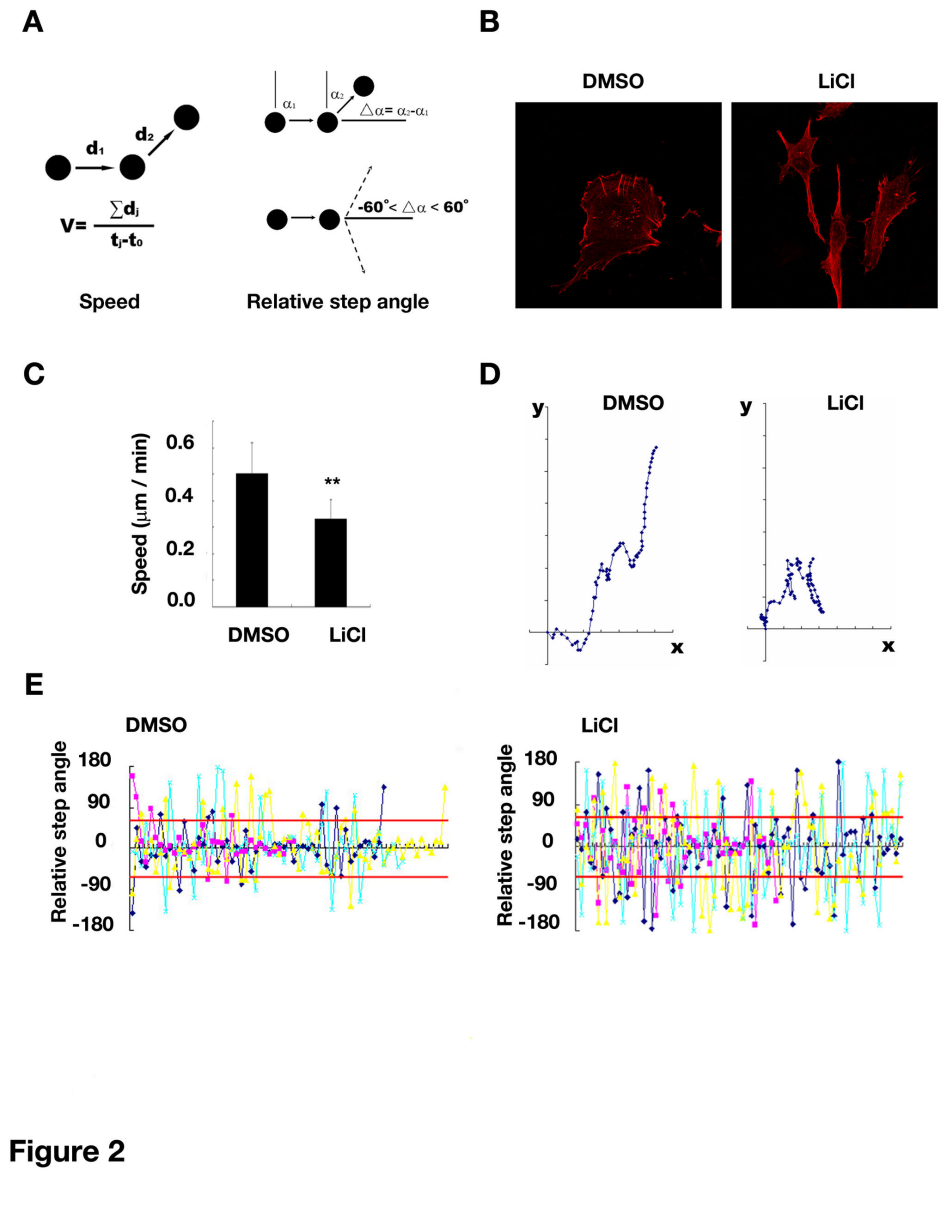
Inhibition of GSK-3 influenced directional persistence and locomotion speed of human glioma cell migration. (A) Scheme to define the speed and relative step angles as described in *Materials and Method*. (B) Morphological changes of the U87 cells treated with LiCl. *Red*: rhodamine phalloidin. *Original magnification, 400×*. (C) Decreased locomotion speed of the U87 cells upon LiCl treatment. Data were mean ± SD of ten cells in each group ** *P*<0.01 versus DMSO. (D) The representative migration traces of the cells in the presence or absence of LiCl, with the starting point of migration superimposed at the origin of the X-Y plot. (E) The relative step angle of representative four migrating cells in the presence or absence of LiCl. Relative step angle and direction change were defined as described in *Materials and Methods*. Negative or positive values in *y-axis* indicated angles in clockwise or counter-clockwise turn, respectively. Red line at 60°or -60° outlined time points when a direction change occurred (beyond the field of -60°<⊿α<60°). LiCl, 20mM.

We next investigated the direct effect of LiCl on glioma cell migration. Through the time lapse imaging of glioma cell migration, we analyzed the morphology change, the directional persistence and locomotion speed of cells as defined in the Methods (Figure 2A). As shown in Figure 2B, migrating cells normally extended a broad, flat, sheet-like lamellipodia towards their moving direction and exhibited a polarized morphology between cell front and rear. In contrast, cells treated with LiCl displayed a multi-polar morphology with several thin and long filopodia, which was comparable with that found in polarity-impaired neurons [14,15]. Therefore, the observed morphological alteration might reflect the disruption of cell polarity caused by GSK-3 inhibition. Moreover, LiCl decreased speed of cell migration compared with DMSO group (LiCl versus DMSO, 0.33±0.07 versus 0.50±0.12 μm/min, *P* < 0.01) (Figure 2C). We next analyzed the directional persistence during cell migration in different groups. According to the motion trail recorded (Figure 2D), the direction change of cells treated by DMSO was gradually accomplished with a small angle change every step, whereas LiCl-treated cells swung in a sharper and more frequent way. When comparing their relative step angles, a parameter indicating directional persistence [16,17], we found that LiCl treatment resulted in a higher directional deviation step by step (Figure 2E). Therefore, inhibiting GSK-3 caused a reduced directional persistence in glioma cell migration. Taken together, these results suggested that when glioma cell polarity was disrupted by GSK-3 inhibitors, the directional persistence and locomotion speed were accordingly reduced. 

### Both GSK-3α and 3β were important for glioma cell invasion

**Figure 3:**
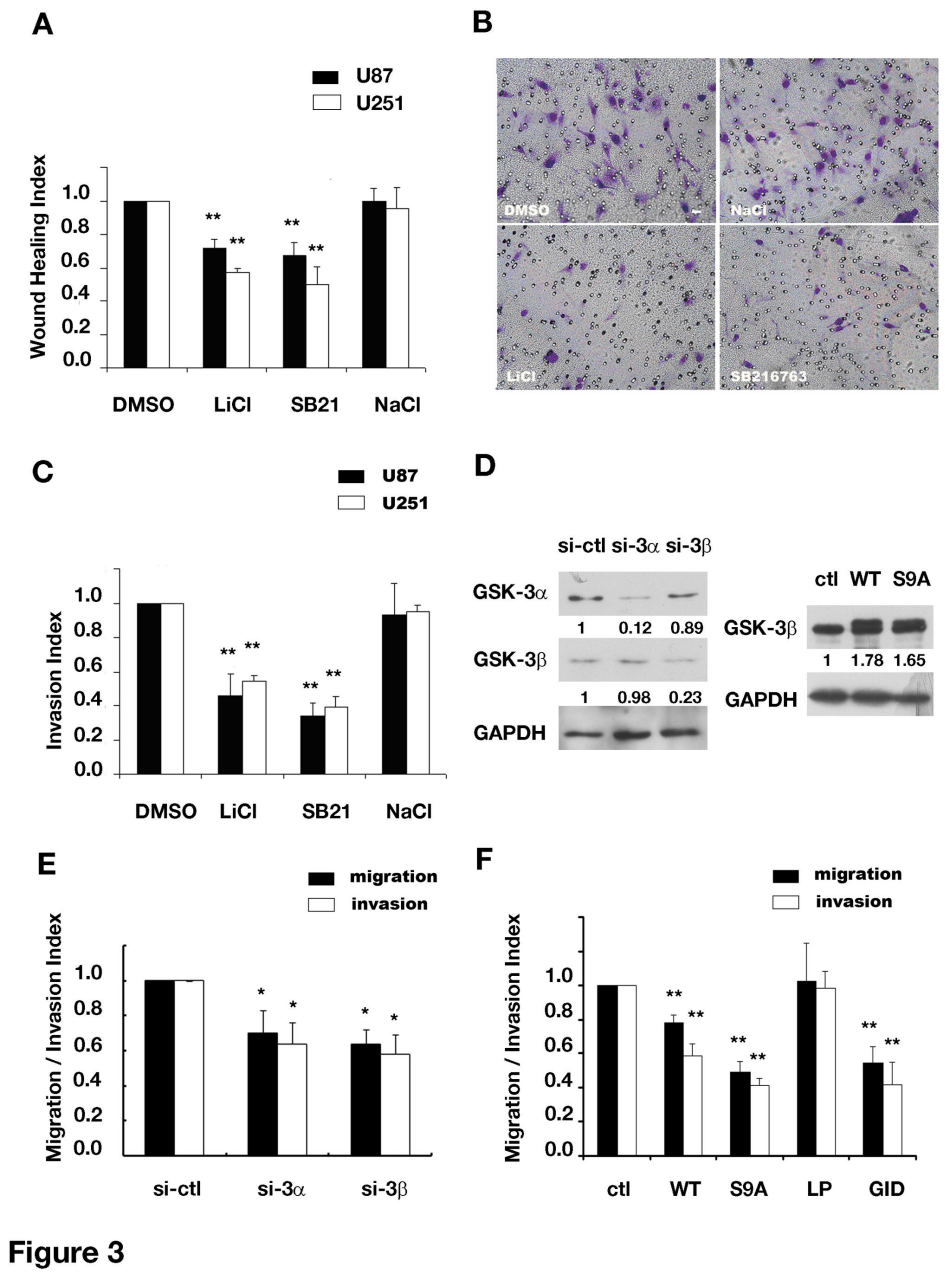
Inhibiting GSK-3 blocked migration and invasion of human glioma cells. (A) Wound recovery of U251 and U87 cells in the presence of indicated agents. ** *P*<0.01 versus DMSO. (B and C) Blockade of cell migration and invasion in the presence of GSK-3 inhibitors. (B) Representative images of U251 cells in the transwell invasion assay in different treatments. *Original magnification, 100×*. (C) Quantifications of transwell migration in invasion assays of U251 and U87 cells. ** *P*<0.01 versus DMSO. (D) Western blot analysis of U87 cell lysates transfected with indicated constructs. The values indicated the amount of indicated proteins normalized to that of GAPDH and compared with non-sense (*si-ctl*) or pcDNA3 (*ctl*) control group. (E) GSK-3 down-regulation by small interfering RNA inhibited U87 cell migration and invasion in the transwell assay. * *P*<0.05 versus non-sense control group (*si-ctl*). (F) Uniform up- or down-regulation of GSK-3β activities inhibited U87 cell migration and invasion in the transwell assay. ** *P*<0.01 versus pcDNA3 (*ctl*). si-3α, siRNA against GSK-3α; si-3β, siRNA against GSK-3β; WT, wild-type form of GSK-3β; S9A, constitutively active form of GSK-3β; GID, inhibitory peptide of GSK-3β; LP, mutant of GID without inhibitory effect. LiCl, 20mM, SB216763, 20μM, NaCl, 20mM. Data were mean ± SD of three independent experiments in triplicate.

We next examined whether GSK-3 affected glioma cell invasion by the wound healing or transwell assays. As shown in Figure 3A, treatment with LiCl or SB216763 slowed down U251 wound closure to nearly half of that in control group and the wound recovery of scratched U87 confluent layer was also sensitive to these inhibitors. As the control, NaCl and DMSO did not affect wound healing. Further analysis of cell viability and cell adhesion demonstrated that these inhibitors did not affect cell survival and cell adhesion (data not shown). These inhibitors also reduced the number of tumor cells invading through the transwell (Figure 3B and 3C). To elucidate the specific role of GSK-3αand 3β in glioma cell invasion, we then knocked down GSK-3αand 3β through small interfering RNA (Figure 3D) and found cell invasion was greatly blocked (Figure 3E). To test the importance of asymmetric regulation of GSK-3β, we uniformly inhibited GSK-3β in U87 cells through transfection with GID5-6, a peptide inhibitor of GSK-3β derived from its interaction domain of axin [18,19], or activated GSK-3β through over-expression its wild type (WT) or constitutively active mutants (S9A), in which the replacement of the Ser9 with alanine made GSK-3βresistant to inhibitory phosphorylation. As shown in Figure 3F, all transfected cells exhibited decreased cell invasion compared with control transfectants. Therefore as the asymmetric distribution of GSK-3β was disrupted by uniform inhibition or activation, glioma cell invasion could be inhibited. Collectively, these results supported that the localized GSK-3β inhibition was important for glioma cell invasion. 

### Asymmetric GSK-3β regulation was important for glioma cell invasion promoted by EGF

**Figure 4:**
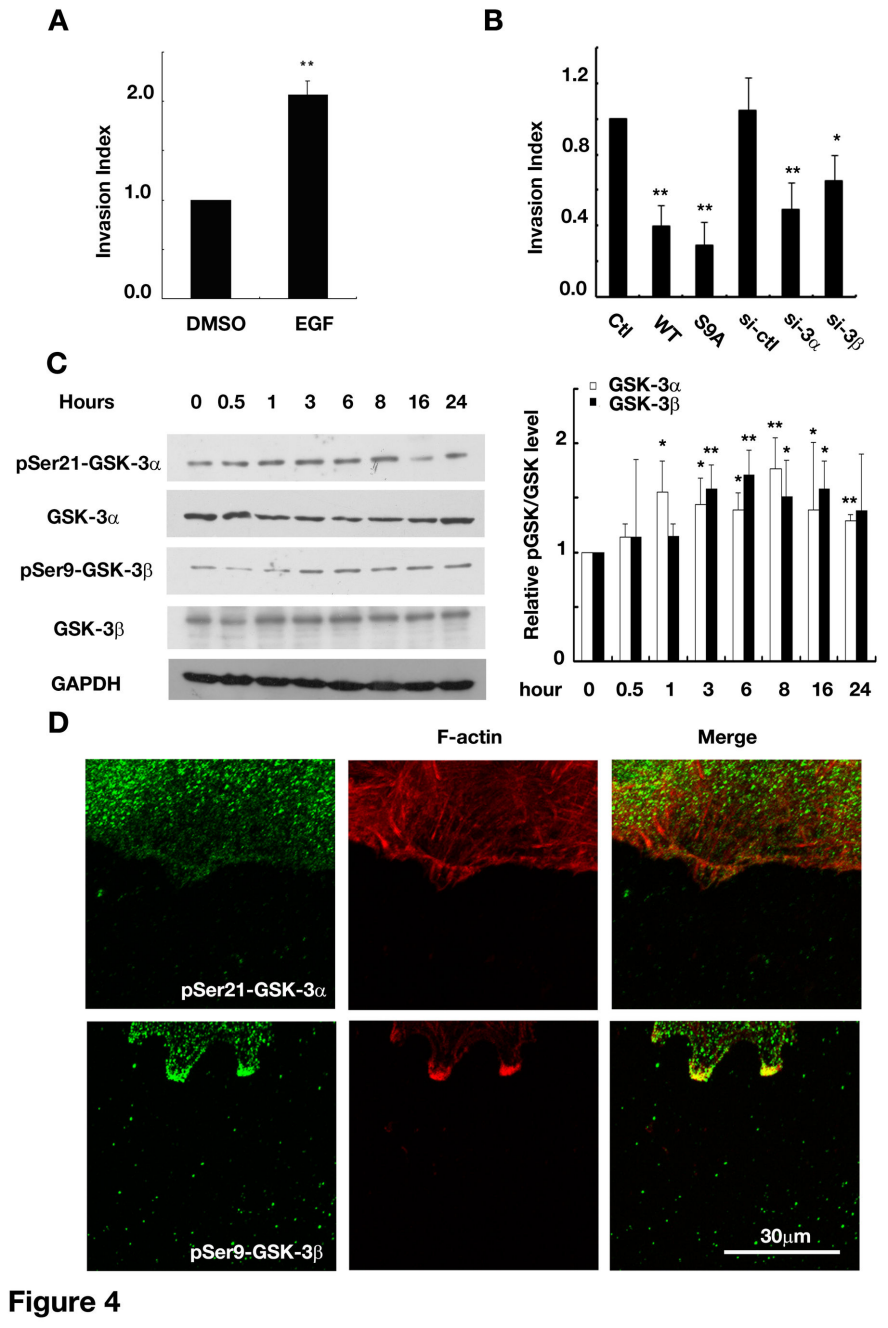
Roles of GSK-3 in EGF-promoted glioma cell invasion. (A) EGF promoted U87 cell invasion in the transwell assay. ** *P*<0.01 versus DMSO .(B) GSK-3 knock-down and uniform up-regulation of GSK-3β activities inhibited U87 cell invasion. * *P*<0.05, ** *P*<0.01 versus pcDNA3 (*ctl*) or non-sense control (*si-ctl*). (C) Up-regulation of p-Ser-GSK-3 levels during EGF-induced wound recovery of U251 cells. *Left*: representative western blots of U251 cell lysates collected at indicated time points after scratching. *Right*: statistics of pSer-GSK-3 levels normalized to GSK-3 levels and then compared with the value at 0hr. *P<0.05, ** *P*<0.01 versus the value of pGSK-3/GSK-3 at 0hr. (D) Location of increased pGSK-3 staining in EGF-induced wound recovery of U251 cells 3 hrs after scratching. *Green*: anti-pSer21-GSK-3αor anti-pSer9-GSK-3β; *Red*: rhodamine phalloidin. *Original magnification, 400×*. si-3α, siRNA against GSK-3α; si-3β, siRNA against GSK-3β; WT, wild-type form of GSK-3β; S9A, constitutively active form of GSK-3β. Data were mean ± SD of three independent experiments. EGF, 40ng/ml.

It has been shown that EGF could greatly promote glioma cell invasion [20]. We then investigated whether GSK-3 and asymmetric GSK-3β regulation were necessary for EGF-induced glioma cell invasion. As shown in Figure 4A, applying EGF greatly promoted U87 invasion. However, knock-down of GSK-3α and 3β by small interfering RNA eliminated the enhancement of cell invasion and over-expressing WT- or S9A-GSK-3β also suppressed glioma cell invasion induced by EGF (Figure 4B). These data suggested that GSK-3 was important for EGF-induced glioma cell invasion and implied GSK-3β might be regulated in an asymmetric manner. Western blot analysis showed that EGF up-regulate phosphorylated GSK-3α and -3β levels in glioma cells upon mechanically scratching (Figure 4C). As shown in Figure 4D, pSer9-GSK-3βwas found mainly among the front lines of the cells towards the wound, meanwhile pSer21-GSK-3α, was again uniformly distributed (Figure 4D). However, the amount and localization of total GSK-3αand 3β was not altered by EGF (data not shown). Taken together, these results suggested that GSK-3α and -3β were important for EGF-induced glioma cell invasion, in which GSK-3β was specifically regulated in an asymmetric way.

### EGF inhibited GSK-3β through atypical PKC and MAPK pathways

**Figure 5:**
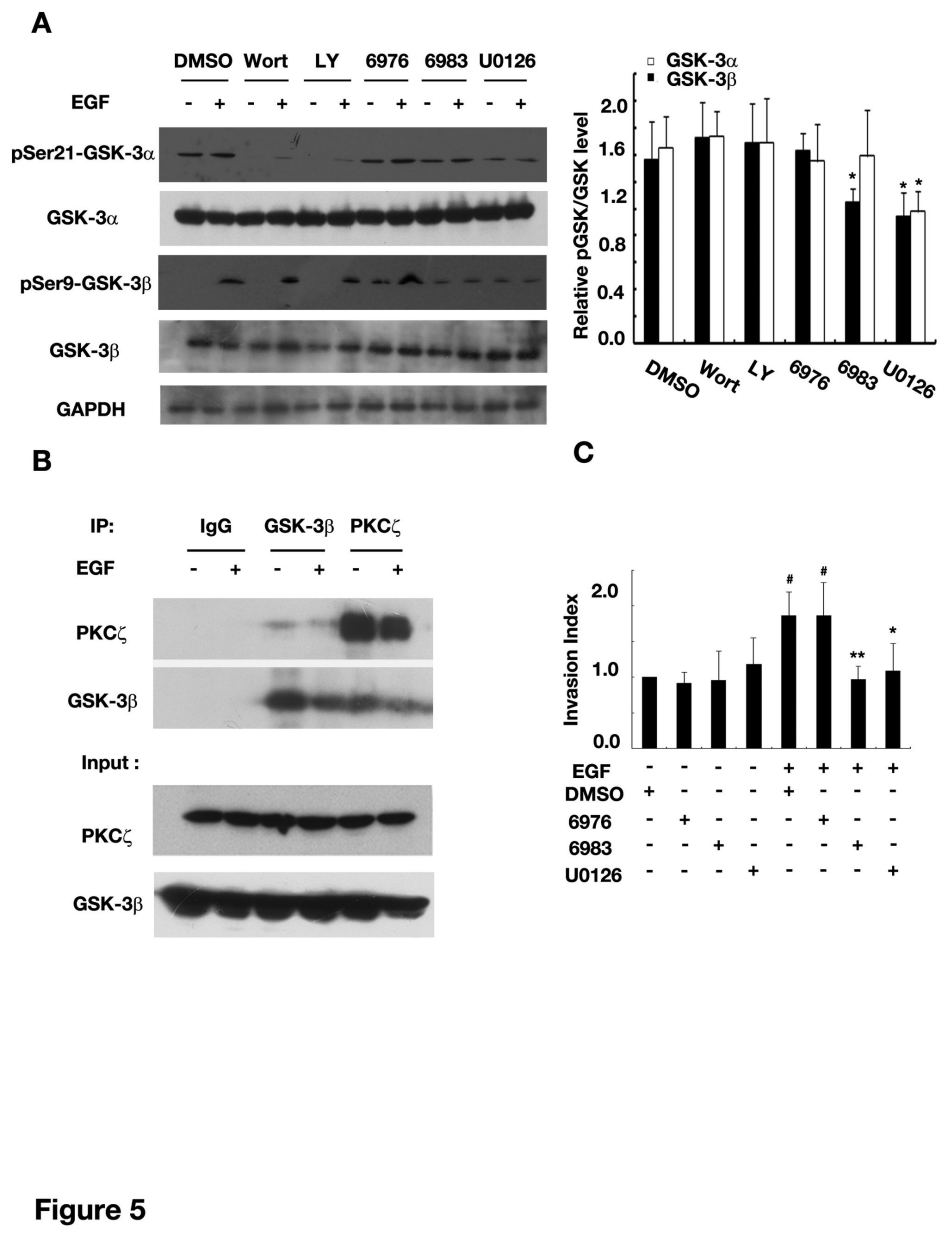
Atypical PKC and MAPK were involved in GSK-3 inactivation in EGF-promoted glioma invasion. (A) EGF-induced increase in pSer-GSK-3 levels was eliminated by inhibitors of aPKC and MAPK. *Left*: representative western blots of U251 lysates collected 3 hrs after wound recovery in the presence of EGF and indicated agents. *Right*: quantification of changes in pSer-GSK-3levels normalized to GSK-3 levels and then compared with the group without EGF treatment. (B) BothGSK-3β and PKCζ were present in the same complexes. Western blot analysis of the complexes precipitated by the indicated antibodies (U251 lysates collected at 2 hrs after EGF stimulation). (C) Inhibition of aPKC and MAPK suppressed U87 cell invasion in the transwell assay. #*P*<0.05 versus DMSO; * *P*<0.05, ** *P*<0.01 versus DMSO+EGF. Wortmannin, 1μM; LY294002, 5μM; Gö6976, 0.1μM; Gö6983, 10μM; U0126, 10μM, EGF, 40ng/ml. Data were mean ± SD of three independent experiments in triplicate.

We then studied the signal pathways responsible for EGF-induced GSK-3 phosphorylation. EGF could induce about 40% increase in pSer9-GSK-3β level 3 hrs after U251 wounded, which was diminished when cells incubated with Gö6983 or U0126, agents known to inhibit pan-PKC or mitogen-activated protein kinase kinase (MAPKK), respectively. In contrast, LY294002 and wortmannin, inhibitors for phosphatidylinositol 3-kinase (PI3K), and Gö6976, the inhibitor for typical PKC, had no effect (Figure 5A and 5B). For GSK-3α, only MEK-1 inhibitor blocked EGF-induced phosphorylation at Ser21 (Figure 5A and 5B). Therefore, in the context of EGF treatment, pSer9-GSK-3βwas likely mediated by atypical PKC and MAPK pathway and pSer21-GSK-3α by MAPK pathway only. It has been reported that PKCζ, a main form of aPKC in glioma cells, could directly phosphorylate GSK-3β [13]. Consistently, PKCζ or GSK-3β were present in the complexes precipitated by anti-GSK-3β or -PKCζ antibodies (Figure 5B). We next examined whether atypical PKC and MAPK pathways were required in EGF-promoted glioma cell invasion. As expected, U0126 and Gö6983, but not Gö6976 greatly eliminated EGF-induced enhancement of cell invasion, and the inhibitory effect was not observed in the absence of EGF (Figure 5C). Therefore, both atypical PKC and MAPK pathways were indeed important for EGF-promoted glioma cell invasion. Collectively, these data indicated that EGF inhibited GSK-3 mainly through atypical PKC and MAPK pathways, which were important for glioma cell invasion.

## Discussion

Although previous studies have revealed that GSK-3β is critical for polarity formation of neurons and glia, its role in glioma invasion was not clear. Moreover, glioma cells infiltrate into the surrounding tissues with a very complicated microenvironment, in which many growth factors, including EGF, play important roles in promoting glioma cell invasion [21]. Here, we reported that GSK-3β was involved in the formation of cell polarity to affect glioma cell invasion, and it was also important for EGF-stimulated glioma cell invasion. Our results suggested that targeting GSK-3β localization might be important for inhibition of glioma invasion [22]. 

Both mobility and direction affect cell migration. We found here that inhibition of GSK-3 suppressed glioma cell migration likely as a result of disruption of polarity formation. This explanation was partially supported by the findings that inhibition of GSK-3 altered the directional persistence and movement in U87 cell migration, characterized by constant changes in direction during cell migration (Figure 4). It is possible that cell polarity was the intracellular clue for direction during cell migration [23,24]. Thus, abnormal cell polarity results in cell migration in a “way lost” manner [25-27]. Another evidence for essential role of GSK-3β-dependent cell polarity in glioma cell migration was shown in Figure 3F. We here compared the migratory ability among glioma cells with up-regulated GSK3β activity by transfecting cells with wild-type or constitutively active forms of GSK3β, as well as down-regulated GSK3β activity by transfecting cells with inhibitory peptide GID5-6. As shown in Figure 3F, both uniformly up- or down-regulating intracellular GSK3β inhibited cell migration. We should notice that all these genetic manipulations would inevitably disrupt polarized GSK3β distribution, either by up- or down-regulating GSK3β activity, thus affecting directional migration. These results suggested the proper intracellular localization of GSK3β activity, in terms of cell polarity, might be more important than the total amount of GSK3β activity.

Although both GSK-3α and 3β activities were regulated by serum and EGF, they likely played different roles in glioma cell migration. Only GSK-3β was asymmetrically regulated at the edge towards which cells would migrate. Therefore, although both GSK-3α and GSK-3β could contribute to cell migration, the localized activity of GSK-3β might play a key role in cell polarity formation. In this context, inhibition of GSK-3βled to changes in directional persistence of cell migration. The differential role of GSK-3α and 3β could be due to their different upstream signals. Although both of them were found affected by the MAPK pathway, GSK-3β was regulated by the atypical PKC pathway, a pathway well known for cell polarity formation [5]. EGF can activate atypical PKC and GSK-3β [28,29], we here showed that EGF induced increase in pSer9-GSK-3β levels in an atypical PKC-dependent, but the PI3K-Akt-independent mechanism. These results were different from the previous report showing that PI3K-Akt pathway mediates EGF-induced activation of atypical PKC [29]. Since in astrocytes the atypical PKC could also affect the levels of pSer9-GSK-3β in a PI3K-Akt-independent manner [13], an alternative pathway to activate atypical PKC might exist in our cultures. 

Recently, EGF and its receptor have been shown as targets for developing efficient treatments against multiple malignant cancers [30,31]. However, glioma has been reported resistant to EGF inhibitors used clinically [30,31], thus alternative strategies against EGF-dependent glioma cell invasion are needed. Our work provided supportive evidences that GSK-3 played a critical role of in glioma cell invasion, especially in EGF-stimulated migration. Although our research did not investigate the downstream signaling of polarized GSK-3β, another work recently identified p190A RhoGAP might be the substrate of GSK-3β required for polarized cell migration [32]. Moreover, lithium has been widely used for anti-depressant in clinical practice [33]. Our findings were supportive for a potential anti-glioma effect of lithium therapy, which has been well demonstrate by other researches recently [34]. 

## Methods

### Materials and cell culture

Antibodies against GSK3β, pSer9-GSK3β were purchased from Cell Signaling, anti-GSK-3α and pSer21-GSK-3α antibodies from SAB, anti-PKCζ and α-tubulin and pericentrin polyclonal antibodies from Santa Cruz Biotechnology Inc., rhodamine phalloidin from Invitrogen, SB216763, wortmannin, LY294002, U0126, Gö6976 and Gö6983 from Calbiochem and the Matrigel from BD Bioscience. All other reagents were from Sangon. The human glioma cell lines, U251 and U87 (ATCC) were cultured in DMEM supplemented with 10% FBS (Hyclone), penicillin (100 units/mL) and streptomycin(100 μg/mL) and grown at 37°C in 5% CO_2_. The small interfering RNA oligonucleotides specifically against human GSK-3α and 3βwere synthesized by GenePharma. The sequences were 5'-GAAGGUUCUCCAGGACAAGTT-3' for GSK-3α, 5’-GUAAUCCACCUCUGGCUACTT-3’ for GSK-3βand 5’-UUCUCCGAACGUGUCACGUTT-3’ for non-sense control. The cells were transfected through Trans Messenger (Qiagen). Cells were harvested 72hrs after transfection.

### Wound healing

Confluent monolayer of U251 and U87 cells was scratched with a 10μl plastic pipette tip after serum starvation for 24hrs, and then was incubated for 24hrs. The width of the wound was quantified under a 200× magnification before and after the wound recovery. The wound healing index was defined as the value of the wound width at 24hrs divided by that at 0hr. Cells treated with DMSO served as the control. For western blot, we performed this assay on confluent cell culture in 100 mm diameter dishes. The wound was made by scraping an 8-channel pipette (with 0.1-2μl tips) several times across the dishes. 

### Migration and invasion assay

The migration assay was performed using a transwell chamber with 8μm pores (Corning). Briefly, starved U251 and U87 (1×10^5^ cells) cells were suspended in 100μl serum-free medium with or without inhibitors as indicated, and placed on the upper chamber for migrating to the lower side of the upper chamber for 16-24hrs. The lower compartment was filled with DMEM containing 10% FBS or 40ng/ml EGF. Then non-migrating cells were removed by wiping the upper side of the membrane, and the cells that migrated to the lower side were stained with crystal violet and counted, and the migration index was expressed as numbers of migrated cells. Cells treated with DMSO served as the control. Procedures of the invasion assay were similar as the migration assay, except that the transwell was coated with Matrigel (BD Bioscience). 

For transfected cells, U87 cells were co-transfected with GFP and the constructs as indicated, in which the ratio of the plasmid amount was 1:3. Cells were collected for transwell assay 48-72hrs after transfection. After tumor cells migrating for 12hrs, the number of total GFP-positive cells was counted, then cells at the upper side were erased, and the number of GFP-positive cells at the lower side was counted as migrated cells. The migration index was defined as the value of number of migrated GFP-positive cells divided by number of overall GFP-positive cells. Cells transfected with empty vectors served as a control. 

### Immunofluorescence

The U251 cells on glass coverslips were washed with ice-cold phosphate buffered saline (PBS) after scratching, fixed in 4% para-formaldehyde (PFA), permeabilized in 0.1% Triton X-100 and blocked in 10% goat serum. The cells were then incubated with primary antibodies and secondary antibodies. Hoechst 33258 (Molecular Probes) was used at 1μg/ml for nuclear staining. Images were taken with a confocal microscope with a CCD camera using LSM imaging (LSM510 Meta; Carl Zeiss MicroImaging Inc.).

Centrosome reorientation was determined as described previously [13]. In brief, U251 cells were fixed and stained with the antibody against pericentrin 8 hrs after wounding. Nuclei were counter-stained by Hoechst 33258. Cells in the first row showing the centrosome located in front of the nucleus and in the 120° sector facing the wound were counted. For each treatment, at least 100 cells from three independent experiments were examined. 

### Immunoprecipitation

Total cell lysates extracted by Nonidet P-40 buffer (137mM NaCl, 10% glycerol, 1% Nonidet P-40, 2mM EDTA, protease inhibitors, 20mM Tris HCl pH 8) were centrifuged at 10,000 g for 10 min to remove the nuclei. Lysates were incubated with indicated antibodies and then washed in Nonidet P-40 buffer. The lysates were then incubated with protein G-Sepharose beads. Immuno-precipitates were collected and washed in Nonidet P-40 buffer and the precipitated proteins were separated by SDS-8-10%PAGE.

### Time lapse imaging

Experiments were conducted in 10mm coverslips that had been coated with 10ng/ml fibronectin (Sigma). Approximately, 10,000 cells were seeded in the normal growth medium and allowed to attach overnight. The cells were then switched to the serum-free medium for 24 hrs to induce quiescence. Cells were then switched to Leibovitz L-15 medium plus 1% FBS (Gibco) and observed using an Olympus inverted phase contrast microscope. A 37°C environment was maintained through the experiments. Mineral oil was overlaid on the medium to provide a sealed environment to prevent evaporation of the medium over the course of the experiment. In a given experiment, images were taken every 10 minutes and single cell migration was tracked for 8-12 hrs. Image data were processed by Image-Pro Plus version 6.0. Speed and relative step angles were used to investigate migration of glioma cells as shown in Figure 2A and previously described [17,18]. The speed (V) represented the total length of the calculated mean path (∑d_j_) of a cell divided by time. Relative step angles (⊿α) of individual cell paths were calculated as relative deviation from each previous step and ranged from 0 (migration in a straight line) to 180 degrees (backward movement), in which ⊿α>0 reflected a clockwise turning while ⊿α<0 reflected a counter-clockwise turning. Direction change was defined if the ⊿α was beyond the field of -60°<⊿α<60°.

### Statistics

Data were presented as mean ± SD of three independent experiments. Statistical analyses were performed with the ANOVA test. *P*<0.05 was considered significant.
